# Geographic mobility and its impact on sexual health and ongoing HIV transmission among migrant latinx men who have sex with men

**DOI:** 10.1016/j.socscimed.2022.115635

**Published:** 2023-01-06

**Authors:** Susan Cassels, Alison Cerezo, Sean C. Reid, David B. Rivera, Colin Loustalot, Dan Meltzer

**Affiliations:** a Department of Geography, University of California, Santa Barbara, USA; b Department of Counseling, Clinical, And School Psychology, University of California, Santa Barbara, USA; c Department of Epidemiology and Biostatistics, University of California, San Francisco, USA

**Keywords:** Sexual minority men, Migration, Social and geographic space

## Abstract

An understudied social process that may determine variable HIV risk, testing, and linkage to care is geographic mobility, including immigration as well as short-term mobility, especially among sexual minority populations. We aimed to assess how geographic mobility over the lifecourse between Latin America and the U.S., and within the U.S., was linked to sexual risk and health behaviors among Latinx migrant men who have sex with men (MSM) in San Bernardino County, California. Qualitative analysis of 16 semi-structured interviews revealed four major domains of influence on participants’ sexual risk behaviors. At the micro level, these included social environment/interpersonal factors (e.g., family and peer support) and geographic factors and pathways (e.g., migration journey to the U.S.). At the macro level data centered on cultural factors (e.g., gender norms in home country) and structural factors (e.g., HIV healthcare). Our results can illuminate and promote effective health policies and HIV reduction efforts for Latinx migrant MSM in metro areas.

## 1. Introduction

In the U.S., gay, bisexual, and other men who have sex with men (MSM) carry a disproportionate HIV disease burden at 59% of new diagnoses in 2017 (Centers for Disease Control and Prevention, 2017; Fauci et al., 2019). Racial and ethnic disparities in HIV prevalence among MSM in the U.S. are startling as well; prevalence and incidence of HIV are significantly higher among Latino MSM than non-Latino MSM (Centers for Disease Control and Prevention, 2017; Millett et al., 2007). Social and structural determinants of health, such as discrimination, physical and social environments, and access to health services, are significant underlying causes of HIV infection and ongoing transmission (Millett et al., 2012; Millett et al., 2006; Wei et al., 2011). For many Latinx MSM, particularly migrant MSM, geographic mobility is associated with sexual migration, which involves forced migration as a means to flee violence and oppression in one’s home country (Cantu, 2009; Toro-Alfonso et al., 2012).

Many sexual minorities are migrating to the U.S., regardless of documentation status, for opportunities to work, to increase their quality of life, and to experience the freedom to express their sexual orientation and gender identity (American Psychological Association, 2019; Cerezo et al., 2014). This change in geographic space often coincides with significant changes in social space as well, such as whether, where, and how individuals access social support, family interactions, sexual health communication or engaging with health promotion services (Diaz, 1997; Shrader et al., 2021). An understudied social process that may determine variable HIV risk, testing, and linkage to care is geographic mobility, especially among Latinx migrant MSM. Geographic mobility, along with the social spaces and contexts of the places that people are exposed to, can significantly affect sexual health practice, communication, and exposure to and uptake of health promotion services, which are essential to curb the HIV epidemic in the U.S.

### 1.1. Purpose statement

The aim of this project was to assess how geographic mobility over the life course between (a) Latin America and the U.S. and (b) within the U.S. was linked to sexual health and HIV risk behaviors among Latinx migrant MSM in San Bernardino County, CA. We were especially interested in exploring the social contexts of origin and destination places, and whether these social spaces were important in shaping the sexual health of migrants. We carried out 16 semi-structured qualitative interviews to explore how migration patterns to the U.S. and within the U.S., and the social environment of these places, impacted Latinx migrant MSM’s social and health experiences, including engagement with HIV healthcare. The overarching goal of this study was to collect data that would illuminate and promote effective health policies for prevention. This work has the capacity to inform HIV reduction efforts for Latinx migrant MSM in U.S. metro areas, more broadly.

This study took place in the greater San Bernardino County, approximately 55 miles east of Los Angeles, CA. We focus on MSM migrants in Southern California metropolitan areas, as urban centers have long attracted sexual minorities (Aldrich, 2004; Weston, 1995). San Bernardino County, CA attracts large numbers of immigrants and internal migrants from Mexico and Central America, and the internal migrant flow from Los Angeles County to San Bernardino County is the largest in/out migration flow in the U.S. Los Angeles County also has the largest total immigrant (or foreign born) population in the U.S. at 3,474, 400 (Institute, 2014). Due to its large Mexican and Central American migrant population at high risk for HIV, San Bernardino County is an ideal location to determine how geographic mobility impacts sexual health and ongoing HIV transmission among migrant MSM.

### 1.2. Conceptual framework

Geographic mobility is a broad term that encompasses any human movement over time and space. In this paper, we focus specifically on two types of mobility: immigration and short-term or internal mobility. Immigration refers to a long-term or permanent move of residence, across an international boundary. For this paper, we refer to mobility within the U.S. as internal mobility. Residential mobility is an internal move of any spatial scale, usually comprising of a change in home or residence. Short-term mobility also encompasses international travel and daily movement. Daily movement is often thought of as activity space, or the places that an individual travels to over the course of a day. The spatial scale of daily movement is variable, limited only by the ability of an individual to move across space within a day.

Migration and geographic mobility are significant social and structural determinants of health and HIV risk. As described in NIH’s Sexual & Gender Minority Health Disparities Framework (National Institutes of Health, 2021), societal, community, interpersonal and individual-level factors must be taken into consideration when assessing how MSM are at high risk of HIV health disparities. In the case of Latinx migrant MSM, migration is an important set of demographic events in the life course; yet, little research has examined how short-term mobility (as opposed to immigration) affects MSM sexual risk behavior, sexual network structure, or risk of HIV transmission (Hernandez et al., 2004).

A rich literature has focused on HIV risk among Mexican migrants to the U.S., finding higher risk among many sub-groups such as labor migrants (Ehrlich et al., 2007; Organista and Kubo, 2005) or individuals living at border regions (Brouwer et al., 2006; Strathdee et al., 2008), and among migrants during different stages of migration (i.e., pre-departure, transit, destination, interception, and return) (Magis--Rodriguez et al., 2009; Martinez-Donate et al., 2015; Sanchez et al., 2012). In addition to individual, contextual, and structural factors, culture has been found to be connected to the elevated risk of HIV for Latinxs. While salient cultural norms, such as familismo, among Latinx culture may foster a supportive environment for Latinxs, other cultural norms, such as machismo, may increase the risk of HIV (Cantu, 2009; Diaz, 1997). Thus, behavioral and physical health outcomes among Latinx immigrant MSM should be analyzed through a critical lens that is culturally attentive and sensitive. Understanding the complex and often nuanced challenges immigrant MSM must negotiate to arrive to the U.S., and while residing in the U.S., will help to reduce HIV disparities.

Increased risk for HIV among migrant populations is a result of individual characteristics of those migrants combined with the broader contextual and structural factors driving migration between Mexico and the U.S. (Kissinger et al., 2012). MSM migrants may be more vulnerable to HIV compared to heterosexual migrants due to greater sexual opportunities combined with social isolation, poverty, limited knowledge of sexual disease transmission and anonymity. Further, sexual minorities experience discrimination within migrant networks that limits access to social and/or economic opportunities associated with positive health outcomes (American Psychological Association, 2019). A lack of social connections to home communities can lead to sexual exploration (Bianchi et al., 2007; Carrillo, 2010; Kobrak et al., 2012; Shedlin et al., 2005). However, destination communities may have a positive influence as well. Research has suggested that neighborhood-level gay presence is positively associated with consistent condom use during anal intercourse (Frye et al., 2010; Koblin et al., 2012). Of course, HIV-positive men (and diagnosed vs. un-diagnosed men) may move for very different reasons, such as seeking a less stigmatizing community or better medical care (Berk et al., 2003; Lieb et al., 2006). By exploring men’s migration histories to the U.S. and within the U.S., including key social and cultural factors, researchers will be better able to understand and address the unique sexual health needs of this community.

## 2. Methods

### 2.1. Participants

Sixteen participants who self-identified as: (a) 18 years old or older, (b) a man who has sex with men, (c) Latinx descent (d) an immigrant to the U.S. from Latin America, and e), currently living in San Bernardino County comprised the sample for the present study. The sample had an average age of 35 years old (range: 23–54). Fourteen participants identified as Mexican, and 2 as El Salvadorian. All participants were cisgender men; 13 identified as gay and 3 as bisexual. Table 1 provides additional information including documentation status and age of migration.

### 2.2. Procedures

#### Key Informant Interviews.

In order to build trust, gain access to the community, and inform our interview guide, we first conducted key informant interviews. Two trained research associates (CL, DM) contacted and visited a number of key individuals working with community organizations, health centers, churches, local bars, and HIV providers in San Bernardino. They then conducted five key informant interviews to explore common discourse around immigration, mobility and HIV risk among Mexican and Central American migrant MSM in San Bernardino. The key informants also provided insight into engaging in culturally responsive recruitment procedures for the target sample, which included the creation of recruitment materials and locations and times to recruit our full MSM sample.

#### Semi-structured Interviews.

Semi-structured interviews were carried out between May and July 2018. All study participants signed a written informed consent. The University of California Santa Barbara Institutional Review Board approved the study protocol. Participants were recruited using flyers posted in venues and community organizations in the larger San Bernardino area identified through the key informant interviews. Potential participants contacted our study coordinator (phone or email). If the participant was eligible, then the semi-structured interview was conducted in-person at a mutually agreed upon location in San Bernardino County. Each interview followed a semi-structured interview guide that asked about residential, social, and sexual neighborhoods or activity spaces, sex, and drug use in difference places; perceptions of places; geo-social hook up apps; health care, HIV/STD testing; childhood neighborhood; immigration to the U.S. and short-term mobility. Interviews were mostly conducted in Spanish (N = 13) and lasted an average 1 h and 13 min. Interviews were audio recorded, transcribed, and translated into English by research assistants at UCSB. Participants received $40 in cash for their participation in the study. Table 1 describes some socio-demographic information from the participants.

### 2.3. Analysis

The overarching goal of data analysis was to identify major themes related to how migration and geographic mobility (to the U.S. and within the U.S.) impacted sexual health and ongoing HIV transmission risk among migrant MSM. Thematic analysis was chosen to analyze the data, a qualitative approach used to identify, analyze, and interpret patterns of meaning in qualitative data (Clarke and Braun, 2017). Prior to the start of analysis, the data analysis team (SC, AC, SR, DR) met over four weekly meetings to review basic theories from our respective disciplines (demography, health geography and applied psychology). The team engaged in assigned readings and discussed each of our approaches to understanding migration, HIV health disparities, Latinx cultural norms, and specifics related to geographic migration in the larger San Bernardino region.

The data analysis team met on a weekly basis to analyze the first interview together. The goal was to ensure we each understood how to navigate the coding software and that we were employing a standardized method for analyzing data. Next, each team member individually analyzed interviews two and three, meeting as a full team to discuss similarities and differences across the groups’ coding patterns. It was at the end of interview three that the team finalized the coding guide for the remaining interviews (4–16). Next, we split our team into two pairs of coders comprised of one faculty member and one graduate student across disciplines (demography and applied psychology), giving each pair half of the remaining interviews to code. The full team continued to meet on a weekly basis to discuss new codes or changes to codes in the coding guide.

**Fig. 1.**
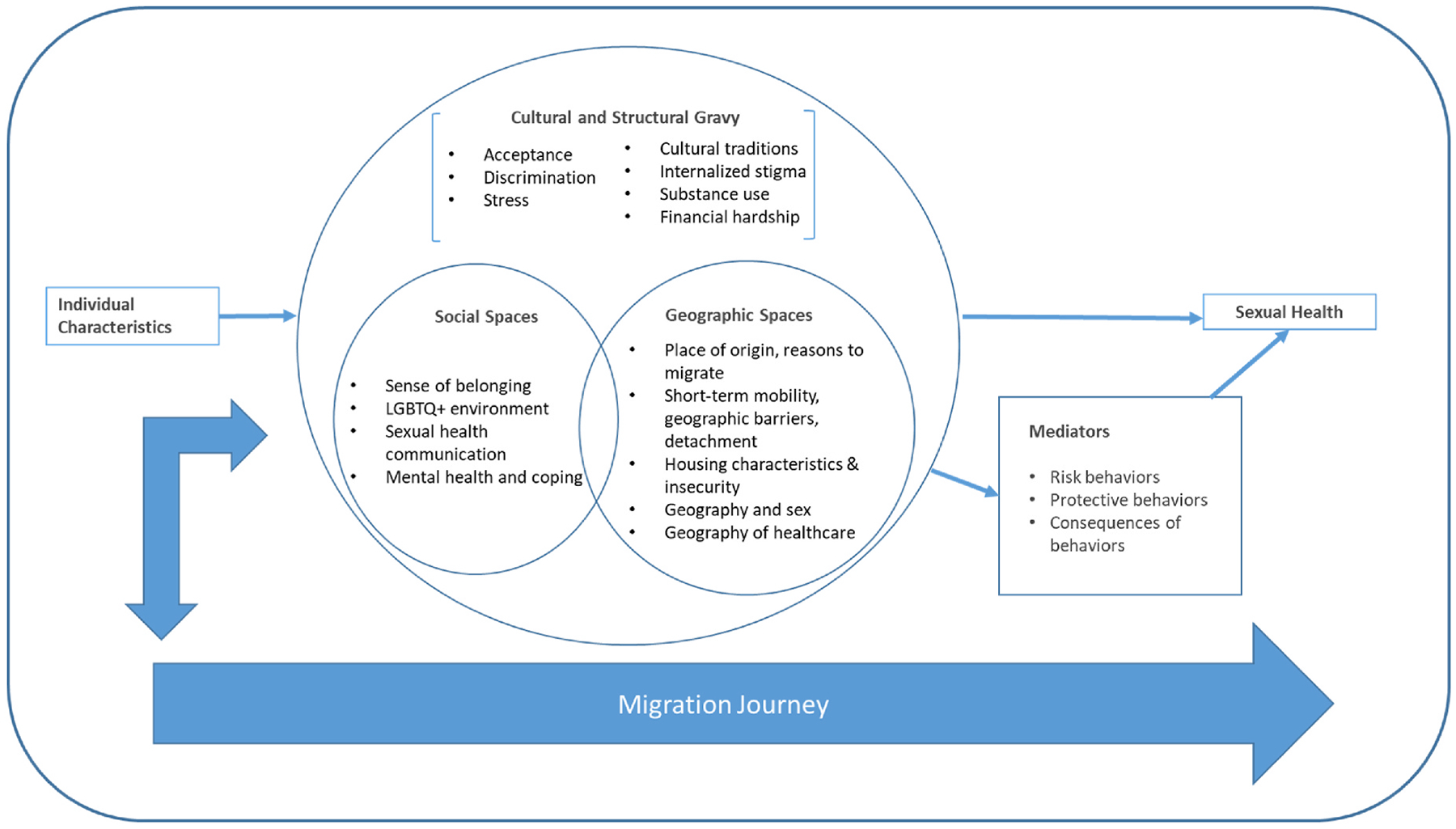
Conceptual framework of geographic mobility and sexual health, resulting from 16 semi-structured interviews of Latinx immigrant MSM in San Bernardino County, CA.

Seventy-three initial codes were identified across the 16 interviews. We removed any codes that were specific to demographic information only (e.g., age, sexual orientation). Next, each pair of coders console-dated the 70 initial codes to reduce redundancy into major groups of themes, noting patterns across the data. This process revealed four major groups of themes, two being central to understanding participants’ direct experiences at the micro level: social spaces and geographical spaces and two that were at the macro level: structural factors and cultural factors (see Fig. 1). Lastly, each team member reviewed all 16 interviews to match quotes to their assigned theme.

## 3. Results

The overarching goal of data analysis was to explore how migration and geographic mobility are drivers of sexual health behaviors and ongoing HIV transmission risk among Latinx migrant MSM. We present the conceptual framework of our findings in Fig. 1. Data revealed that geographical spaces, the physical spaces MSM reside and live in, and social spaces, how MSM build social community and the social norms within these communities, were the most salient in MSM’s sexual health behaviors and access to health supports. However, the drivers and consequences of geographic and social spaces on sexual health overlap, hence the intersection in Fig. 1. We also found that the larger macro system – cultural and structural factors – were key in explaining men’s sexual health. We conceptualized these factors as “gravy” because they were the connective elements that linked the major themes to tell a cohesive story about sexual health risk. This is the main component of Fig. 1; however, we situate these factors along a pathway, linking individual characteristics to sexual health outcomes. The geographic and social spaces exposures can both directly affect sexual health outcomes, such as providing safe access to healthcare services, and indirectly via mediating factors, such as a need for anonymity leading to higher risk sex. Lastly, all of these factors are interacting with the migrant’s journey over time, with bi-directional effects.

We report the geographic space themes from our qualitative analysis first, and then the social space themes second, with relevant cultural and structural themes (identified in *italics*) embedded in the two sections. For the most part, quotations are listed in the table in the same order that they are presented in the results. Key exceptions include cultural and structural themes that cross-over between geographic and social spaces.

### 3.1. Geographic mobility

Geographic space can affect sexual health, both directly via neighborhood exposures for example, and indirectly by amplifying the consequences of exposures or limiting or eliminating behavioral choices. Early life experiences and exposures can have immediate as well as long-lasting implications for health later in life. Patterns of geographic mobility determine exposures to different social and physical environments, and can impact sexual health, risk behaviors, and access to care. Activity spaces, or the places that people live, work, and socialize, are also important determinants of health. Thus, we examined respondents’ experiences and exposures in their childhood homes, during their migration journey, recent short-term mobility, the places where they currently live and socialize, as well as their geographic spaces of sex.

#### 3.1.1. Place of origin, reasons to immigrate

Out of 16 respondents currently living in San Bernardino County, 13 were born in Mexico, two in El Salvador, and one in Texas who moved to Mexico around the age of three months. The respondents had mixed emotions about childhood and their childhood homes. Many mentioned that they loved their neighborhoods, and that their place of origin was beautiful, or provided a good education (Table 2: Quotes #1, 2). However, several mentioned significant violence in their place of origin. Some said that they did not feel safe due to their sexual orientation or HIV status, and these fears featured prominently as a reason to move to the U.S. (#3–6). “*That is was not my place*.” “*Being gay there is hard*.” Negative childhood exposures, both physical and emotional, are known to have long lasting impacts on health later in life. Many of the participants in our sample had unstable childhood experiences, feelings of not belonging, and experienced violence and trauma, all of which are considered acute childhood exposures that can negatively affect adult health.

Participants discussed how cultural norms directly or indirectly related to their well-being and geographic mobility. *Acceptance* and *discrimination* were emergent cultural themes driving migration decisions. Multiple participants described migrating from their home country because the U.S. was perceived as being more accepting and respectful of one’s sexual identity in comparison to their country of origin (#72). Participants often decided to migrate from their country of origin to avoid sexual orientation discrimination, such as abuse and bullying (#4). Experiences of discrimination occurred in multiple forms (e.g., physical enactments, verbal expressions, bullying, etc.) early in the life span. Most experiences were related to aspects of one’s identity as a sexual minority, or as an individual perceived to be living with HIV (#67–72). The discriminatory actions and comments often came from members of one’s family and/or within their country of origin, often because participants did not conform to traditional gender roles. Some participants also described the role that their geographic location and environment, such as being around family members when residing in their country of origin, affected their *mental health* status often as a result of sexual identity discrimination/bullying (#91–94).

*Financial hardship* emerged as an important structural factor many participants faced in both their origin and destination locations. While participants primarily cited cultural and social reasons for their decision to migrate to the U.S., they also discussed financial insecurity in their home country (#7, 8) related to discrimination or lack of formal education. Due to these drivers of migration from their home countries, many participants moved to the United States with limited resources available to them. In the absence of resources like legal help or financial assistance, many participants faced difficulties related to documentation status in the United States.

Documentation status served as primary driver of *financial hardship* because participants in the study were not able to find consistent work that paid a living wage. Participants cited being unable to find work or the work they had did not pay enough money for their basic needs (#9). The available jobs often lacked access to health insurance, which served to further drive financial instability (#10). It is important to note that financial hardship that influenced the decision to migrate from the origin was often still a problem to contend with in the United States for many of the participants in this study.

#### 3.1.2. Short-term mobility, geographic barriers, detachment

At the time of the study, the duration that participants had lived in the U.S. ranged from 2 months to 32 years, and the age of move ranged widely (see Table 1). About half of the respondents said they have never gone back “home”, or have not left the U.S. at all, and about half report that they go back to visit family, friends, partners, and some to get health care (#11). The cultural theme of *acceptance* appeared to be related to return migration as well. A couple of participants added that one motivator of migrating or not returning to their country of origin was that people who are living with HIV are often more accepted in the U.S. (#74).

Participants reported high levels of short-term mobility, both residential mobility as well as daily movement. One participant said, “*I can’t stay in one place, and then I need to move*.” Some of the reason for short-term mobility was because San Bernardino does not have the health or social services that participants needed, or places to socialize (#12, 13). Additionally, they pointed out an incongruence in residential, social, and sexual places. Many respondents mentioned that whenever possible, they go elsewhere to socialize (more on this below, in geography and sex section).

Even though participants had diverse activity spaces, issues of transportation and geographic barriers were prominent. Geographic barriers also confounded social isolation; many participants mentioned that they did have friends to socialize with, but that they lived in different places (#14, 15). Taking the bus, traffic, travel time all affected the types of activities, places to live, and ability to socialize (#16).

Similar to other research on activity spaces for sexual minority men, the participants expressed lack of attachment with their places of residence that contributed to feelings of isolation. For some participants, anonymity in their residential neighborhood was expressed as a positive characteristic (#17–19). Many described their residential neighborhood as calm, or expressed that their current place of residence was an improvement on the previous place. However, many participants described their home as simply where they sleep, and did not express much meaning or attachment to the place or the people (#20, 21). Two respondents mentioned that they plan to move as soon as they are able (#21, 22). A number of participants mentioned that they specifically avoid engaging with others in their neighborhood as a safety precaution (#23, 24), although one participant explicitly expressed that their neighbors watch out for one another.

Participants commonly disclosed experiencing *stress* related to insecurity around geographic mobility, residential spaces, and living situations (#25–28). For instance, one participant described witnessing traumatic events in a shelter for houseless individuals when in Mexico (#26), others expressed stress regarding neighborhood violence (#28). Experiencing financial stress in order to obtain basic necessities (e.g., food, housing, etc.) was also reported (#25).

#### 3.1.3. Housing characteristics, housing insecurity

Housing instability and homelessness were major themes among the participants as well. Housing insecurity is a major reason for high rates of mobility among this population, and can lead to trauma, loss of autonomy, higher risk sexual contexts, and lack of access to prevention and health care. Multiple respondents mentioned unstable living situations (#29–31), temporary and transitional housing, and homelessness (#32–34).

Respondents had very diverse living situations, including living with parents, other family, roommates, or living by themselves. Positive experiences with housing tended to align with comfortable housing co-habitants (#35). Many more participants, however, mentioned discontent with their living situations and lack of privacy, and that these challenges affected their wellbeing (#36, 37). One participant said, “*I think it is because I do not have my own space. I do not have my own space. I do not feel psychologically fine*.” A number of participants expressed the desire to move, to acquire their own place to live if possible (#38–40).

Many of the participants found themselves in undesirable residential locations based on what they could afford (#41). These locations were distant from potential work locations and required more time commitment to travel. This exacerbated *financial hardship* and made it more difficult to find and keep employment. Differences in social and family support paired with financial hardship drove differences in housing stability among participants. Participants that lacked family support relied on social services to alleviate housing insecurity (#42, 43). The housing services played a key role in placing individuals into some form of housing to avoid periods of homelessness. The participants primarily cited being connected to housing services through mobile testing centers, HIV specific healthcare clinics, and other community health organizations. They cited not knowing about these services until they were advertised at those locations, which points towards these services and organizations playing a key role in several aspects of the overall well-being of the people they serve (#44).

#### 3.1.4. Geography and sex

Similar to other research on the geography of sex for sexual minority men, the respondents’ activity spaces were incongruent. Participants mentioned the need to find safe places to socialize and engage in sexual activities, and these spaces were usually separate from residential spaces. Many participants expressed hesitation to bring sexual partners to their home residence. Sometimes this was because of a lack of privacy or housing characteristics (#45), and other times it was due to fear, stigma, or lack of safety in their residential neighborhoods (#46–48). Participants occasionally reported engaging in sex at home, but only when privacy was guaranteed or they hid from roommates (#49). Therefore, if sex at home was either not desirable or not an option at all, respondents mentioned traveling to other spaces/places to engage in sex (#50).

Respondents also mentioned that they were hesitant or unlikely to look for new sexual partners in their residential neighborhoods. Sometimes this was due to the previous concept that San Bernardino may lack gay-friendly spaces. Sometimes it had to do with geographic barriers or stigma/fear, discrimination or fear of violence, and preferring anonymity (#51, 52).

Geo-social hookup apps were common among the sample of respondents, and many had strong feelings toward whether or how the apps should be used. These apps supported and reinforced separation of residential, social, and sexual places. Apps were associated with the geography of sex, including looking for sex in new places, or at time of first arrival in a new place, and made seeking a new partner easier (#53). Apps may also allow people to safely find sexual partners when they otherwise do not feel comfortable publicly seeking a partner (#54). App use appeared to allow individuals to go to different places to seek specific types of partners or engage in specific types of sex. One respondent in particular talked about how using apps and engaging in sex was a type of escapism – engaging in potentially risky sex “behind closed doors” because they did not see a future in themselves. Nonetheless, even though use of geo-social hookup apps was quite common, respondents voiced many conflicting views (#55, 56), and some felt that app use could lead to harm.

#### 3.1.5. Geography of healthcare

Participants in our sample, both HIV negative and positive, consistently cited access to healthcare as a primary concern for their well-being. For many, the primary barrier was their documentation status and the host of difficulties associated with it. The participants in the study cited challenges related to health insurance being tied to certain types of jobs. Lack of documentation also served as a barrier from receiving public benefits related to healthcare needs. These two barriers most often resulted in participants having no insurance and elevated the cost of care to unsustainable levels (#57, 58).

Differences in healthcare systems between the origin and destination locations played a role in expectations and interactions with the healthcare system in the U.S. For example, some participants discussed the differences in pay structure between the U.S. and their home country where in their country of origin, they only paid for the medicine they were prescribed and did not have to worry about copays or other consultation related fees, which was unexpected for them. Based on the additional costs incurred for receiving healthcare in the U.S. without insurance, some participants opted to return to their origin country to receive medical care. While this practice reduced cost of care, it extends time between visits and limits access to healthcare in time of emergency.

Among participants with access to healthcare resources, adequacy of care was a concern. Participants discussed the difficulty of finding providers who addressed their healthcare needs appropriately. They described doctors who were disinterested or uncomfortable with them (#59) or that participants themselves felt uncomfortable talking to doctors about their unique healthcare needs. At times, communication was not possible due to language barriers with doctors and staff (#60, 61). Some participants were able to utilize translators to communicate to their doctors and some had doctors that spoke Spanish themselves, but these resources were not available to everyone and resulted in differentials in healthcare quality. Many of the issues described above were from general care doctors or clinics that were not associated with HIV treatment and testing. Conversely, fewer challenges were present with care situated in LGBT communities (#62). Doctors were often bilingual and costs associated with preventative care and testing were free of charge. The doctors were also specifically trained and equipped to work with patients who have HIV specific needs and created an environment where the participants felt more comfortable being open and honest (#61). Participants cited that they were referred to HIV specific care from their primary care doctors or resources from the LGBT community-based resources in their area (#63).

Participants in the sample also described a geographic disconnect between their residence and their healthcare needs. Clinics and hospitals often were located outside of their residential neighborhoods (#64) and the distance between their homes and healthcare facilities was large. Pairing the distance with transportation infrastructure in the San Bernardino area, where participants must deal with traffic congestion and unreliable access to transportation, resulted in travel to healthcare appointments being highly time consuming. The time commitment associated with healthcare visits was a barrier to regular care and resulted in missed appointments or not scheduling appointments at all due to other time commitments (#65).

### 3.2. Social spaces

Social spaces describe the mechanisms by which MSM in our study built social community and came to understand their social position within the U.S. related to migration, race and sexual identity. Further, social spaces played a key role in shaping MSM’s sexual health experiences, particularly related to forming social norms for how and/or whether to engage in sexual health communication and safe sex practices. Several subthemes emerged in the larger domain of social spaces, which included sense of belonging, internalized stigma, LGBTQ + environment and sexual health communication. Men’s cultural backgrounds appeared to shape the results as well, mainly cultural expectations related to gender roles within Latinx culture.

#### 3.2.1. Sense of belonging

Several respondents endorsed “sense of belonging” as an important factor in their lives as Latinx immigrant MSM. Some participants noted that being MSM often drove them to keep their lives private (#66) and in some cases, seek solitude as a means to live authentically (#67). This included fueling migration to the U.S. to seek refuge from *cultural traditions* rooted in misogyny and patriarchy that made it challenging for MSM to live an authentic life in their home country (#68). Participants shared having to suppress their sexual identity and gender presentation to avoid being targeted by others (#68–70), including ongoing pressure from friends and/or family to uphold gendered expectations (#71, 72). Finally, men also discussed the pressures they faced with living with HIV, which included facing stigma as an HIV + man (#73, 74). It is important to note that for some participants, negotiating identity and gendered norms was a challenge that extended into the U.S. post migration, particularly within Latinx enclaves or when they resided near family members (#75). As noted in “housing instability” and “geography and sex” sections of the present paper, cultural pressures extended beyond social needs to directly impact men’s residential and sex location choices.

Some participants reported that negative messages about MSM they heard from an early age, both within their families and home communities, contributed to *internalized stigma* about their own gender presentation and sexual behavior, “*hey, this guy is a guy and should behave like a guy*.” Participants also noted learning how to present as heterosexual, “*It’s more like formal. My head up. Jeans, pants, t-shirt. Like mask myself so they don’t say anything*.” Participants shared how the importance of “passing” as heterosexual was necessary to avoid discrimination and violence (#76, 77). An important factor to consider with respect to internalized stigma was that because men sought out social spaces where they could live more authentically, they looked to the U.S. LGBTQ + social scene as the ideal norm from which they learned how to live as MSM, including how to dress and engage in safe sex communication and/or sexual encounters.

#### 3.2.2. LGBTQ + environment

Participants described the LGBTQ + environment as including both physical spaces where MSM gathered and online communities, primarily dating apps, where men could find social and sexual partners. Men shared that in both physical and virtual spaces, there were significant pressures to look a certain way related to weight, clothing and other markers of success (#78, 79). Further, men noted that although the process of finding sexual partners online was easy, there was no trust that sexual partners were being honest about their HIV status (#80–82). This included either brief or no mention of HIV status and other sexual transmitted illnesses—a trend that shaped their own engagement on apps. Further, men noted that finding meaningful connections online was difficult, including heavy representation of heterosexual MSM on dating apps (#83).

#### 3.2.3. Sexual health communication

As noted by several participants, the U.S. LGBTQ + social scene, including online spaces, set the tone for how to engage in sexual health communication. Participants noted that with some sexual encounters there was no communication related to HIV status (#84) but that the use of condoms was an expected norm (#85). Although sexual health communication did not always happen with casual encounters, some men found supportive communities via groups within social service spaces—namely Latinx LGBTQ + organizations—where they could discuss and learn about sexual health (#86–89).

Social services were identified as a key community site among participants, playing an important role in connecting men to services and care they needed and were, at times, unaware of. Specifically, many study participants noted mobile HIV testing centers. Some mobile testing centers run by community-based health organizations provided services in Spanish, and were often the only source of testing or HIV specific care that some respondents were willing to use. The information provided to participants at these mobile testing locations was key in connecting them to other services as well, such as clinics and housing services. Further, mobile testing locations helped bring care to where participants were located, serving as an entry point into a larger network of care to improve men’s health and overall wellbeing (#86–90). Before being referred to or informed of these services, many participants were not aware these services existed or thought they did not meet the eligibility criteria to use the services.

#### 3.2.4. Mental health and coping

Mental health and coping was a major theme shared among the sample. This theme falls under the larger umbrella of “social spaces” in that most participants’ mental health was rooted in challenging interpersonal relations, including access to adaptive coping outlets. Participants shared bouts of depression related to lifelong experiences of discrimination (#91, 92), including rejection from family (#93). Some participants noted how mental health challenges arose when they became HIV+, related to fear and shame (#94). A major source of stress and coping was engagement in *substance use* and for some, it became a driver of financial hardship (#95). Participants noted how addiction permeated into all areas of their lives through financial hardship that strained their living situations (#96). Some men noted that use of drugs during sex, as in “party and play”, was an activity they learned in the U. S. (#97, 98). This included partying for several days on end to escape their home life, often with older, non-Latinx men who had the means to provide drugs, housing and financial assistance (#99). In one case, a participant shared that a sexual partner revealed that they were HIV + only after several days of using drugs and having sex with them.

With respect to mental health and coping, participants also disclosed how they prioritized their well-being or engaged in activities that were good for their mental health. For instance, one participant described that journaling and attending support groups were helpful strategies when navigating through finding out they were living with HIV (#100). Further, some participants shared how being outdoors was “therapeutic” when they sought alone time and privacy (#101) and they learned how to set boundaries to protect their mental health (#102). Another source of positive mental health was support from one’s family upon disclosure of one’s sexual orientation. As described by one participant, “*That’s why I came out … it was more important for my parents to accept and respect it*.” And, as noted by another participant’s mother upon his disclosure, “*Well, what I can do is love you equally as I do with your siblings*.”

## 4. Discussion

The overarching goal of the present study was to explore how geographic mobility—to the U.S. and within the U.S.—influenced sexual health and risk of HIV transmission among Latinx immigrant MSM in San Bernardino, CA. The findings of the present study, with respect to drivers of migration to the U.S., are consistent with extant research (Abreu et al., 2020; Cerezo et al., 2014; Gonzalez et al., 2021). Namely, most participants shared that the decision to migrate to the U.S. was rooted in their perceived opportunity to be more open about their gender and sexual identity and to do so in a manner that would not bring about violence or employment discrimination. In addition to social and cultural motivation for migration, many participants also cited potential for improvement in economic, occupational, and educational opportunities. Again, this finding is consistent with previous research on LGBTQ + migrants (Cerezo et al., 2014; Gonzalez et al., 2021).

The findings from the present study make important contributions to the literature on sexual minority migrants and health. Many participants shared how sexual activity, and norms related to sexual health, took place in distinct geographic areas that were separate from their residential location. With respect to residential location in the U.S., participants noted having to negotiate ongoing heterosexist cultural norms and lack of acceptance from family and others living in their immediate residential community. This resulted in residential isolation where participants were detached and uninvested in their place of residence and simply viewed it as a place to sleep rather than as a home. Some participants reported having to travel to more distant locations to find and participate in affirmative social and cultural spaces, including spending significant time and transportation resources, which were at times difficult given ongoing financial limitations. Due to the infrequent visits to desirable locations, participants relied on hookup apps to find sexual partners and/or social companions, which was coupled with risky sexual encounters and/or substance use. However, it is important to highlight positive experiences that emerged from the data for this underserved population, as they illuminate areas where social service and health policy efforts should be augmented in order to improve the mental and physical well-being of Latinx immigrant MSM. A few participants indicated that their current living space was an improvement when compared to their previous living space, described their current home as “calm”, and used housing support social services to avoid being homeless.

### 4.1. Employment and healthcare considerations

Another important finding was that employment opportunities and healthcare needs were often geographically disconnected and distant for participants. The participants in our sample stated that when searching for employment, they were faced with low wages in their immediate area or had to contend with long and difficult commutes to reach jobs that paid higher wages. Having reliable transportation in the form of a car or public transportation were cited as the primary barriers to finding and holding a better job that is located further from their place of residence. Other structural variables, like documentation status, also limited the occupational opportunities.

Healthcare resources were also noted as distant and inaccessible to participants. Clinics that accepted participants’ insurance or offered affordable healthcare providers were not near their residential location and required them to traverse longer distances, which again required reliable transportation. The participants cited that their primary healthcare providers did not speak the same language as them, which made communication of important healthcare needs difficult. They also cited feeling uncomfortable talking about HIV related healthcare needs with their primary care doctors and were often referred to HIV specific clinics, which added another node in the growing network of places needed to sustain basic healthcare needs. Nonetheless, participants tended to have more affirming experiences in HIV-specific care often because of welcoming environments, having translators or bilingual providers, and working with providers who were understanding of their unique needs and experiences. Expanding affirming healthcare in non-HIV-specific settings for Latinx immigrant MSM may increase engage of health services in primary care settings with a focus on preventative health.

### 4.2. Isolation in residential neighborhoods

Participants in our study shared experiences of social isolation within their residential neighborhoods, often Latinx immigrant enclaves. These experiences were linked to sexual risk, including using hookup apps to find sexual and social outlets or seeking out areas like West Hollywood, CA and Palm Springs, CA where participants could maintain anonymity. It is important to point out that MSM seemed to rely on these outside areas as the settings in which they learned social and sexual norms, as in whether and how to discuss HIV risk and safe sex practices. Participants shared how these lessons were often misaligned with safe sex and instead involved “party and play” as the primary means of forming social connections and improving men’s sexual encounters. These findings demonstrate the need for immigrant MSM to have healthy, anonymous outlets within their residential communities, even in the form of online social groups where men can learn about social norms, HIV risk and treatment, and how to connect to social services that will aid in meeting their basic needs. Although some men sought out anonymity and privacy related to sexual orientation, critical resources to reduce HIV transmission within men’s immediate residential location are important and should be widely available. This might include advertisements on hookup apps, on public transportation, in general medical offices and in other areas that immigrant MSM frequently come into contact with (Cassels et al., 2020; Yang et al., 2016).

### 4.3. Cultural considerations

The pressure to meet familial and cultural expectations related to gender roles and masculinity was widely described by the sample. Nonconformity with traditional gender roles resulted in discrimination, bullying, and stigmatization among a majority of the participants in the sample and was the leading factor in men’s decisions to migrate to the U. S. Several men shared how they were pressured to have children, engage in romantic relationships with women and for two men in the sample, maintained long-term relationships with married men who succumbed to Latinx gender role pressures to marry women. These pressures were connected to men’s internalization of negative messages related to the LGBTQ community, including heteronormative ideas related to how men should dress, speak, move and be in community with other men. Health providers should be cognizant of the cultural pressures many Latinx MSM face and how such pressures might serve as a barrier to seeking out HIV care. Further, as shared by several participants, many Latinx MSM are in long-term committed relationships with women, including marriage. It is therefore critical that providers not assume the sexual behaviors of immigrant men and instead ask open-ended questions related to the past and present sexual partners outside of their stated sexual identity and/or long-term relationship.

### 4.4. Limitations

Our sample was comprised of 16 Latinx immigrant sexual minority men in San Bernardino County. It is important to note that the majority of the sample was of Mexican descent. Given the differing socio-political contexts in Mexico versus Central and South America with respect to migration and LGBTQ + rights, the results presented in this paper are reflective of Mexican immigrant MSM and therefore should not be used to understand all Latinx immigrant MSM. Our team spent considerable time working and building relationships in the San Bernardino community, and this allowed us to recruit men that fit our eligibility criteria. Many of the respondents were recruited through connections with community leaders and social service organizations. Therefore, Latinx immigrant MSM men who were less connected to the local community, or who were not living openly as a sexual minority individual, would have been less likely to be recruited in our sample. Our study took place in 2018, before the COVID-19 pandemic. Geographic mobility and its connections with sexual health and HIV prevention may be different now, since COVID-19 had a significant impact on immigration and short-term mobility. Lastly, the relationship between geographic mobility and HIV risk is most likely non-linear over time; the period immediately after migration to a new city is a time of heightened vulnerability, and risk seems to peek 1–5 years after the move (Egan et al., 2011; Lama et al., 2015). Our sample included men who had been living in the US from 2 months to 30 years. We were unable to assess specifically how the timing of immigration was associated with many of the themes that we identified. Additionally, three participants moved to the U.S. before age 15, thus the decision to migrate was potentially not theirs to make. It should be noted that these participants discussed their choice to remain in the US as connected to having a better quality of life as related their sexual orientation and/or HIV status than would be possible in their country of origin.

### 4.5. Policy recommendations

Data from the present study made clear the key role that community-based health organizations and social services play in educating Latinx immigrant MSM about HIV as well as connecting them to basic resources like housing and employment assistance. Federal, state and local funds should be used to bolster and expand these services. Health interventions should be considered from a geographic lens to take place in the areas where the riskiest behaviors take place. A key example is the use of mobile HIV testing vans by local Latinx community health organizations that provided crucial healthcare needs and information on available social programs. It is also important to note that transportation was a major barrier to HIV care. Community-based health services should be provided with transportation resources like bus vouchers, gas cards and carpools that can help ensure men can seek HIV care within their local communities. The findings from the present study illuminate barriers encountered by undocumented Latinx immigrant MSM in accessing and obtaining healthcare. Expanding federal and state level policies to grant affordable physical and mental healthcare to undocumented Latinx MSM may diminish existing health disparities in the U.S. While certain state level policies, such as California’s Medical young adult and older adult expansion program, have attempted to address the healthcare access barrier for undocumented individuals, certain restrictions continue to prevent a large portion of the undocumented community from accessing affordable healthcare. Within the context of STI risk and transmission, the lack of access to affordable healthcare may pose a public health concern given that Latinx immigrant MSM may not receive preventative care, such as PrEP, STI testing, and HPV vaccines, highlighting the need for policy efforts to be inclusive of this population. Due to multiple intersecting identities (e.g., sexual minority identity, undocumented, etc.), this population requires their identities be protected in order to ensure safety and uptake of services. Therefore, policies and programs that aim to support this population should prioritize securing anonymity.

## 5. Conclusion

The complex geographic network connecting the social and cultural aspects of immigrant Latinx MSM underscores the importance of geographic and culturally circumscribed health interventions and policies. The results from this study highlight the importance of considering accessibility and time spent at highlighted areas of intervention to ensure successful connection with the most vulnerable MSM. Geographically circumscribed interventions show promising improvements but disparities still exist and continue to grow among Latinx MSM. The consideration of complex geographic mobility patterns and temporal dynamics discussed by the participants deserve greater research attention to close the disparity gap in new HIV infections.

## Figures and Tables

**Table 1:** Socio-demographic characteristics of study participants (n = 16).

ID	Age group	Place of Birth	Age group of permanent move to US	Duration living in US (at time of interview in 2018)	HIV status	Documentation status	Sexuality
1	20–24	Mexico	10–14	13 years	negative	temporary protected status	gay
2	25–29	Central America	20–24	2 years	positive	documented	gay
3	30–34	Mexico	30–34	2 months	positive	unknown	gay
4	30–34	Mexico	30–34	2 years	positive	undocumented	gay
5	50–54	Mexico	20–24	30 years	positive	temporary protected status	gay
6	30–34	Mexico	15–19	14 years	positive	temporary protected status	gay
7	40–44	Mexico	20–24	22 years	positive	unknown	bisexual
8	25–29	U.S. (Moved to Mexico age 3 mo)	5–9	18 years	positive	citizen	gay
9	20–24	Mexico	15–19	5 years	positive	unknown	gay
10	50–54	Mexico	20–24	32 years	positive	documented	gay
11	35–39	Central America	20–24	14 years	negative	undocumented	bisexual
12	20–24	Mexico	0–4	23 years	positive	unknown	gay
13	30–34	Mexico	15–19	16 years	negative	temporary protected status	gay
14	35–39	Mexico	20–24	12 years	negative	unknown	bisexual
15	45–49	Mexico	30–34	18 years	positive	unknown	gay
16	50–54	Mexico	20–24	30 years	positive	documented	gay

**Table 2:** List of key quotations by 16 Latinx migrant MSM participants, by theme.

Quote number	Theme	Participant ID
	*Subtheme*	
**Geographic Spaces**		
*Place of origin, reasons to migrate*		
1	In my childhood it was a really nice place, but I knew that it was not where I wanted to be my whole life. Because specifically, my persona, with the risk of bullying, a lot of bullying, and my sexual behavior, and with my sexual orientation. A gay person in a town is not very well accepted. There is a lot of bullying in occasions, it was never my case but there were people that received physical abuse even, and a lot of emotional and psychological abuse. So, it was not for me, I think that when I had the opportunity at my 17 years of age, when I had the use of reason, I decided to leave my town.	5
2	I wasn’t very happy. I love [place of origin], I love my city of [place of origin]. But, over there I lived … I have a society that is misogynistic. But I … that does not take away with the love I have for the place. It is a really beautiful place and that is all. With … a lot of education.	8
3	Okay, well one of the reasons I made that decision to come to the United States is because I feel like there is more respect. First, for being gay, for being a homosexual, there is a lot of respect. Second, for my disease.	3
4	The neighborhood was kind of, um, like, not racist, but kind of …. towards gay people. They were really not okay with them … there were a few people that used to lived there that were gay. They used to get bullied at lot.	1
5	Because it is a difficult city to be in. There are no openings for homosexual people like myself.	4
6	And being gay there [place of origin] is hard. Because they think that the man is for the woman and the woman is for the man. So, there is a lot of discrimination	9
7	And from the [city in Mexico] I came to the United States for the economic situation. Well, living in [city in Mexico] is very nice but living there is really expensive and the salaries are real low. You can’t live there	5
8	It was nice, it was nice, everything was nice. When I decided to come here, it was to work and to send my mom money because we weren’t so well off financially in Mexico. So we came here. I came here with a brother that lived in [city]. And I came to the United States.	11
9	Um, but since I, at the time, well, still working on it, did not have papers, because I was brought illegally here when I was a little kid. Um, I couldn’t go to school.. or get proper healthcare … [after high school] Yeah, that’s when I started realizing how hard it was getting medical attention. Um, any other kind of going to schooling or going to college.	1
10	So he didn’t have Medical. Um, when they took him to the hospital. We were basically going to pay for the ambulance, the hospital, every single treatment that they did at the hospital. And we weren’t able to afford it- not even the ride of the ambulance, it was like $11,000.	1
*Short-term mobility/geographic barriers/detachment*		
11	No, I go to Mexico. I don’t have a medical card; I don’t have insurance.	14
12	I guess [residential neighborhood] still really like close to- there’s no gay bars, there’s no- nothing here. So, it’s like, the only gay bar or restaurant near is all the way into [city approximately 30 miles away]. And that’s the only one, other than I need to travel all the way to [city approximately 70 miles away] or LA to find something like that. So, I can at least be comfortable.	1
13	Yeah, I had to travel to [city in Mexico] to go to the clubs and all. No, when they open up a new bar it only lasts like for months. There are not enough people, it is better to go to [city in Mexico] and have fun. Because they have clubs and it’s a bigger city, so it’s better to travel to [another state] and party or to [city approximately 110 miles away]. Because it is very conservative, I mean nobody likes to be pointed and say, ‘oh you’re gay’.	14
14	Um, but all my other friends that I had in high school- they all live in [city]. And sometimes I do talk to them, and I do make plans to go see them, but it’s just hard. Because it’s like without traffic, it takes me an hour and a half.	1
15	He stays 45–50 min away from my apartment, he is the only person that I talk to, but he doesn’t stay nearby. So, I mean sometimes we just meet in LA, somewhere else where we can just like drive halfway. But yes, he is the only person that I can consider like my friend. Uhm, his family, I do have communication with his family, but like from my family I have nobody, no brother, no sister, nobody.	9
16	So … it was hard for us, you know? I mean, I had to take the bus, like today, I had to take the bus to the clinic, you know? It’s … the whole traveling thing, you know? It’s like I had to … like all that travel had me … “oh I don’t wanna go because I have to do all this to get there” but I know that it’s for my own good, it’s for my own health, it’s for my well-being, to live a little … to live longer, to adapt to this lifestyle, I have to do what I got to do, you know? It’s not just gonna get given.	14
17	I felt good because I did not talk to anyone, it was only like a ‘hi’ and ‘bye’ and all, and that us what I liked—to be in my own world, to no bother anyone or to be bothered.	8
18	Yes, I felt comfortable. I felt like I fit in because no one ever disrespected me and yes, I did fit in. Yes, yes it was better. It was better than the previous place.	5
19	Everyone is in their apartments– their lives. Everyone has their lives there. And when there is a birthday or a meeting. You know who your neighbor is. They will not tell you how you are. I am sometimes and I go out to smoke. And some that go out with their dogs. I greet them, “Good morning.” But no. Everyone has their life. I like it.	2
20	Um, no, no, like I said, I am not so close to the neighborhood because I always make friends that are outside of the neighborhood.	15
21	In whichever opportunity I am going to leave, I am going to do it.	8
22	At this point of my life, it doesn’t matter where I live. Yeah, I don’t care. Cause right now I am just saving up money cause I want to move to a different neighborhood.	14
23	because the neighborhood that I live in-well everyone looks normal, they’re married, husbands, wives, there isn’t guy to guy, female to female. So yes, it would affect it. But I do not feel comfortable to take a person to my house being a guy because they are going to think that I am gay. And I do not want to create that image. Because I do not like it.	6
24	I don’t have any friends ….I do not talk to the surrounding neighbors, I, I don’t know. I am always very careful with that because often times you make friends, then there are problems. That’s why everyone is better off in their own thing.	11
25	It’s always been hard to find like a clinic … that will take me. Until a few weeks ago, a month ago, I guess, when we found out we could get food stamps. Because there was a period of time that my husband wasn’t getting disability. So that was really hard because there was no income coming. And we needed to pay for food, we needed to pay for rent, the car, insurance, phones. It was really hard.	1
26	Yes, it was something horrible. I was traumatized from what I saw. I was left with traumas … inside the shelter. I saw lots of bad things. But all thanks to god, I came out physically fine. But there were some really ugly things in there.	3
27	Right now, I am going through a pretty difficult situation. This is because I am practically living with nothing where I am currently being lent a room. Right now, I am asking for help from a department to try to obtain my own space. Because right now where I am at, no … But I feel like it is all little by little. Well, the beginning is always hard. Many people might go through what I am going through, and they might say “oh no, I don’t like this”. And well, no, because it is all little by little. So, the most important thing is that I am being treated well for being gay and for being a person with HIV. And that is what makes the United States be a first world country and what makes its mindset be entirely distinct from the countries in Latino America. They do it in a distinct manner. They are inclusive.	3
28	A bit dangerous because you always hear gunshots. Gunshots and well, the people from around already know me. And well, they know that I do not get into it with anyone. And well, in all forms, there are lots of gunshot accidents.	8
*Housing characteristics, housing insecurity*		
29	Different. For example, last year I was in this place, and the other year I was in another place. Different places. Different cities	2
30	I have been living with friends, from house to house, and I have been moving constantly.	4
31	I live there because I have no other option, I think that if I had another option to live in another place I would do it.	6
32	That is difficult. I–when I moved to [city] I moved alone. I have never told my parents this, but I had nowhere to go … I moved to a shelter. I only lived there for two weeks. Yes, it was something horrible. I was traumatized from what I saw. I was left with traumas.	3
33	Yes, I was … I was, I was like, like a year, a year more or less, or almost the year it was some months … But I did live … well in the street exactly, no. I lived sometimes with friends.	11
34	Okay, one of the reasons of why I left the house that I used to live with my uncles was out of fear. It was when … Yeah. I was scared when I was diagnosed with HIV—of being able to contaminate my cousins and my uncles. Because my uncles were my two uncles and they had 4 kids.	6
35	Where I live right now … its better … I am better … I feel more comfortable, gay guys live there as well, ‘roommates’. And … they are all very friendly, there aren’t any problems about anything.	15
36	I think it is because I do not have my own space. I do not have my own space. I do not feel psychologically fine.	3
37	I do not have … privacy. No, it is my own room, but they do not let me take guests. No friends, nothing.	12
38	I do not have the funds to say, ‘I am going to another place’, I am also not being charged a lot where I currently live.	12
39	Right now, I am just renting with my cousins, and my uncle and my aunt. So, I am very controlled.	14
40	Ahm, no, it is only temporary, I want to get my own apartment, I want to be alone, I want to have my own place …	15
41	Um, well for the time being, I do not have the funds to say ‘I am going to another place’, I am also not being charged a lot where I currently live. But if I found a better place, maybe, yes. Where I could be [more] comfortable.	12
42	it’s hard because my family does have money, but not really. And then since I cannot, um, cannot lean on them- on that. Me and my husband do not make that much money. Since he’s not working because, um, he just got [illness], we found out two years ago	1
43	Right now I am going through a pretty difficult situation. This is because I am practically living naked where I am currently being lent a room. Right now I am asking for help from a department to try to obtain my own space. Because right now where I am at, no … But I feel like it is all little by little	3
44	Because … I … heard in the HIV support group that I used to go to, where I would go, I heard that there was a program with housing in part with the [type of] church …. … So, I asked and they told me yes … so I told them that if I could get housing and they told me the requisites that I had to sign, and they told me that if they had a room they would contact me and … like almost within the 5 days they told me that I could go live with them if I wanted to.	11
*Geography and sex*		
45	I was, I guess I was 18 on my first hookup. Like, all the guys that used to text me and send me messages through the app would tell me to, like, go somewhere or to meet them up. But I wouldn’t because I was scared or, you know? I didn’t know what they would do. So, I always, like, tried to keep it at home, like somewhere safe that would be. But most of them, I wouldn’t - I wouldn’t actually bring them home. Because - or just wanted to do that. Or at the time, I was living with my family. I– I shared my room with my mom. So, it’s like I couldn’t do anything.	1
46	Want to have sex with somebody, I go to the place, I go to the motel, I go outside, and outside can make everyplace, but not my place. A place is for rest, for living, for you know, enjoy my time,	16
47	But first, I would not do it with anyone from my neighborhood. I would not do it because to me it is very dangerous. They could say … from there they could get opportunities to hurt someone.	8
48	No, I don’t like to do it in the community, in my neighborhood, like it’s um, it’s kinda awkward, you know? It’s not awkward, but it’s kinda like um, it’s just like the one rule you don’t do, like you know, in your neighborhood,	13
49	He went to the room, to my room … we snuck in.	12
50	But I … yeah, La Quinta, or Quality Inn, I mean they’re nasty old motels but … hey, it’s a good place to spend some time with no rules and just having fun, you know?	13
51	Because I don’t like it then looking for my neighbors, or my … somebody close to me … you know, I don’t like it when you have a relation in your same work. If you get relation with somebody, it’s complicated cause you stuck, or it’s too much close and then … sometimes you want, like okay, I have a relation with somebody, but I have my independent life.	16
52	Before yes, before I preferred … I preferred to go far, like I said, I go where I feel free, where no one could be like “Oh, I know him”, “oh, he’s here”, “oh, him” so like I have never liked it when they make that type of comment. I have never liked it.	15
53	When I used that application, when you go to a different place, it was easier to connect, to meet people in the app. Because you are a new face, someone new to the city, or to that place. And it is a bit … it’s really easy to have sex when you move to a new place where it is not your neighborhood.	15
54	I would not go out in the streets looking for it. It was through an app … there is how I was able to get in contact with those people.	4
55	Well … truthfully the gay community is very vulnerable to using those applications. Um, and a lot of times there could be people that are bad people that can cause harm through an application.	4
56	Yeah, those apps they’re just riding exclusivity within men, young men. Especially when the community is like that, It’s like you know, it’s not a good idea. Those apps are poisoning people’s minds.	13
*Geography of healthcare*		
57	Um, but since I, at the time, well, still working onit, did not have papers, because I was brought illegally here when I was a little kid. Um, I couldn’t go to school.. or get proper healthcare … [after high school] Yeah, that’s when I started realizing how hard it was getting medical attention.	1
58	There are some barriers on not being a resident or citizen of this country brings barriers to not being able to get the proper medical attention. For example, if you appear from a chronic disease, like HIV, it’s more difficult because you have to pay off of your own tab and it is more difficult getting medical insurance for people that are like in my situation.	4
59	You have to ‘click’ with the doctor …. I feel it is because they do not understand. They do not know much about you. The doctor should know a bit of psychology …. But I feel like something is missing, something is missing from the doctors and that is psychology, to listen to the patient …. I think the doctor should listen to the patient speak about all parts of their lives—because the life of a human being, yours, mine, and that of whichever person is based on what you have lived in your life ….That is what is fundamental and the doctors do not ask that. Instead, they always ask—“What hurts?”, “here?”, “oh, okay, take this medication” and there. It should not be like that	3
60	Because, she does not speak Spanish. I need a doctor who speaks Spanish.	2
61	Um, like, um, I have always had Kaiser, ha-ha. Like I like Kaiser because like I feel like the doctor like, well, my doctor always asks me ‘have you had sex? Um, you need to do these studies’. They are always focused on me, it’s not like other clinics …. That I have heard of like ‘they didn’t let me talk’ or something like that. My doctor speaks spanish, so that is a great advantage because I tell her ‘this hurts’ or ‘could you check this out’ or something	10
62	Yeah. For the most part, yes, always. If you go to a place within the gay community, it is more proabable that you will get the care that you want	15
63	Uhm, it’s through friends, normally if you have friends within the community, they are the ones that can guide you because … you can go to the internet and search but … it is not … like how a friend would tell you, “oh, I’ll take you there”, or “I’ll take you over here where I go normally”	15
64	In my neighborhood, no. There is one close to my neighborhood that is in [city]	6
65	Like right now, it’s been a barrier. Especially after [injury] and … I had mobility problems, the barrier was transportation. And the other barrier is that sometimes economic, I don’t have an income.	16
**Social environment and interpersonal factors**		
*Sense of belonging*		
66	I guess I never felt like I fit in with other people, like always tried to. Like kinda like be friendly, um, be always happy, smiling but like in me, I never felt that way. I always felt like I was never fully, thoroughly myself. There was only half of me people were seeing.	1
67	I liked it [living in Mexico], but only to a certain point because I was never able to be who I am. I had to pretend or appear to be a person that I was not. I had to be macho as well. I did not want to because my sexual preference is not oriented to what they wanted me to be … so people started telling me things, bullying and more bullying, and they criticized me a lot and that was a part of my life that scarred my life.	4
68	And … I suffered a lot of bullying as a kid. I suffered bullying because … uhm, it was when I was a kid, I was like a girl … my gestures, my way of being was like a girl … so, people started telling me things, bullying and more bullying, and the criticized me a lot and that was a part of my life that scarred my life. When I was a kid. Since I was a kid until adolescence, up until I was a teenager it was very difficult for me.	4
69	So, I know that if I say that I am HIV positive, I will get fired from my job, so I would lose many things. The same with my family, I know that perhaps one or another person might understand but I know that no. I know that no. I know them.	6
70	They would basically make fun of me all the time. They would call me princess or girl, or things like that, so like the bullied me.	10
71	I was like more into guys like I’m gay accept it, I’m gay. In Mexico, you know they just judge you; you know prejudice because religious, their beliefs are like ‘you’re not supposed to be with other guys’. You’re supposed to have girlfriends, get married, and raise a family.	14
72	When I used to live in [state], I used to like always try to like, let’s say I couldn’t wear a pink shirt … or very tight, um pants, because I was like, “Why? They are going to think I am, like they are going to point at me and be like ‘ey, you’re gay’ because you are wearing skinny jeans.” Now I can wear whatever I want.	13
73	… because with them … they weren’t well informed about HIV, aids, and all that, and I am a carrier of HIV, so they weren’t well informed on that and … there were some things that I didn’t like because they would separate my plates, my cups, and I told her “it doesn’t transmit like that” … and I would explain and explain that it can only transmit only by sexual intercourse and blood transfusions.	11
74	Well, actually I do not think that I will go back because I am diagnosed with HIV. I am gay. So, I think that it would be a great hit to my family that they would not say. I come from people that are very misogynistic, and that is something that is like, it is like an end, so like, it is like a no. So, no. Currently I am in the process of fixing my status, I have my work permit. But no, I do not see myself in [city in Mexico], I see myself here.	6
75	… My life is calm, uhm, a bit difficult because again I’ll say … I have to be living as though I was a macho, a man, and I don’t want to be like how people want me to be. I want to be who I am, and despite being in a country where people have an ‘open mind’ it is difficult regardless, because it’s a thing for Hispanic people, Latinos, to not accept gay people.	4
76	I saw how they suffered in [city], I went to some gay discos … I said, what I am going to do is … so what I saw in other friends was that they were masculine, they were very masculine, and they lived with their partners, and I would say, I could … I want to become like them. Be masculine.	10
77	After I came out, it’s just something you need to access like you need to behave, like for me like being so feminine is like being like a clown, I was raised that way. I don’t know why, but that is the way I think.	13
*LGBTQ* + *environment*		
78	Even person or people that I know is basically through apps, websites, online. And even though that’s making it easier, for us to date, um, it’s really hard. And not, it’s funny though, because I feel like gay people are really, like, really, like … I don’t want to say like, um, they bully a lot of our, like, more than just straight people. They are really like picky, really, like, they’ll tell you, like “I’m not- you’re not what I’m looking for, like don’t talk to me.	1
79	The community asks for a lot, in the way that you dress, how you look, what your race is, what type of race are you … within the gay community, they will always see you like how you look, your appearance has a lot to do with it. And when you go to a straight place, no one worries about you on how you look, or what you have on.	14
80	I’m doing the everything that it takes for me to take care of you and I think if everybody or the gay community um, throughout this gay application, social media, they can just be real, just respect themselves, just like I did, like it hurts, it takes a lot of time for you to admit like ‘hey, I’m HIV positive.	8
81	I’m not just looking for sex, especially in the gay community is really hard to actually trust somebody and even maybe trust your partner.	8
82	Regularly when you meet a stranger on Grindr, you can ask about the status, you can ask them about STDs or about HIV, but I think that it is a dumb question because they aren’t going to tell you the truth. People are going to say “Oh, I have HIV”. “I have STDs” no, no one, that’s a dumb question … because no one will say the truth, they are strangers.	14
83	Most of the people that texted me, they were straight men, usually with wives or girlfriends. Um, I would tell them no because I wanted to be with someone that would actually put me first, that won’t hide me, that when you came down to something, they’ll be there for me.	1
*Sexual health communication*		
84	I don’t know, I felt like it was just for fun being over there, but I just realized, like all these guys that could have been HIV positive, and they didn’t tell me anything.	12
85	No, no we did not talk about anything because it was simply just sex. So, like there was no time to talk about anything. It was a nice time, but that was all. Always using condom, always.	6
86	Through the Foundation …… They told me where to go and all.	3
87	I am volunterring in various centers in which, I only have one—it is called ____, so I know that ____ is one of the biggest centers here in California. And that one way or another, they can help me, because, well it does not seem very difficult to find help, so like I do not have problems.	6
88	No, no, I took it because … I didn’t have symptoms, I took it one time when I went to ____, and I was doing it with a friend, we went … we passed by there and they told us “come guys, we are offering a HIV test, it’s free” and well, then I got in … and …	11
89	When I move in to LA to … to Fontana, San Bernardino, sorry to the … uhm, San Bernardino, I’m starting to coming and I go to ____ … and he referred to me the … county department, the health department	16
90	Until a few weeks ago, a month ago, I guess, when we found out we could get food stamps. Because there was a period of time that my husband wasn’t getting disability. So that was really hard, because there was no income coming.	1
*Mental health and coping*		
91	And that is how years pass for many. But yes, they have been made very difficult for me. I feel psychologically bad. I have not had much peace where I could say, “Oh, this is where I always feel at peace and in tranquility. I could live these next years in tranquility”. I have never had that.	3
92	Yes, I wanted to come here for everything. For the life I brought … I suffered from depression because of the discrimination I was chubby, and I was in the house.	2
93	Um, depressed because basically my whole family on my dad side did not support me because they were very religious. They would tell me that no, that I had to go to church, so they would make me feel bad for being gay. I basically felt bad all the time … since 15 years old …	10
94	Um, and she was the one who was with me when no one else knew I was HIV positive, so she saw me crying, suicidal all these things that were going through my mind. And she even told me she … and now-a-days she’s really easy to talk about the topic, I was like ‘yeah’ like it’s not as hard as it is.	9
95	I was making $900 to $1200 a week you know and then I lost it all because you know, I fell in love with the wrong person you know, and then I fell into their habit and then just, it was just a big ol’ comedown … there’s nothing good about those substances	13
96	When I didn’t have a place to live, that was when I started hanging out with people that consumed drugs	11
97	You would have had to experiment and have those types of friends to be able to know, but when you are out of that circle of people that are getting drugged, you do not know what drug is the one that is being consumed the most there	15
98	So, it’s kinda weird, like hooking up, just all that time, it’s kinda just like poisoning my mind about that.	13
99	I used to really like the casino too, I was addicted to that place too. Yeah, doing drugs and not wanting to go have sex with all of them [dates] to maintain a little bit of sanity, I took them to the casino, and at times I would win money, you know?	13
100	I always carry a notebook and a pencil and sometimes I write on how I feel, I write it, I write it and there, when I finish, I destroy it and there—that was like a therapy for me so I think it is like a feedback of my own, in which sometimes I do not see things, well I grab a mirror and I stare at it and I try to see the good and bad of me, so I think that it has worked because I am, without looking for support groups, without anything, simply my own self. I have a desire to be able to go out and say ‘I have this and this and this’, but I do not do it, definitely not right now because the people do not understand me.	6
101	I really like the sound of the cars and the city, but when I want to feel alone, I like to do those types of things, to the rural areas—where it is not so populated and where there aren’t so many people … I like to feel that the same when it rains, I like the smell of wet rain. It makes me definitely feel good. That is why I go there. It, it makes me feel like therapy, including when I was diagnosed, I think that it helped me a lot.	6
102	Okay, because in the past, I spend too much and share too much my life for help other ones, And I forget a little bit of myself I don’t forget completely, you know? But I am forgetting, and I am very busy with the lives of other ones, and I forget my life … I stay and work or … go to the bar, blah, blah, blah, but it’s not the same because all of the time, somebody calls me “ey, you can help me?“, “I help you.”	16

## Data Availability

The data that has been used is confidential.
